# Editorial: Advances in social constructionism and its implications for career development

**DOI:** 10.3389/fpsyg.2024.1485935

**Published:** 2024-12-10

**Authors:** Peter Yang, Charles P. Chen

**Affiliations:** ^1^Department of Counseling, National Chiayi University, Chiayi, Taiwan; ^2^Department of Applied Psychology and Human Development, Ontario Institute for Studies in Education, University of Toronto, Toronto, ON, Canada

**Keywords:** career construction, career identity, constructivism, dialogue, meaning-making, narrative, reflection, social constructionist perspectives

## 1 Introduction

The evolution of social-constructivist and constructionist paradigms in career development can be traced back to Collin and Young's (1986) groundbreaking work. Published in the prestigious journal Human Relations, their work elaborated on the constructivist paradigm, challenging the mainstream approaches to career counseling. Following Collin and Young's seminal work, Mark L. Savickas emerged as another fundamental figure in promoting social constructionist thought in career development. In the early 1990s, Savickas advocated the integration of narrative approaches into career counseling, which led to the development of the career construction theory (CCT). In this theory, in order to explore the dynamic and evolving nature of career development, Savickas (2005) highlighted how individuals make sense of their careers through stories. Around the same time period, R. Vance Peavy's contributions became highly influential in constructivist counseling. His socio-dynamic counseling provided a practical approach to meaning-making and explaining the interplay between personal agency and environmental influences and its influence on career development (Peavy, 2000). The early 1990s also marked the formation of the social cognitive career theory (SCCT), developed by Robert W. Lent, Steven D. Brown, and Gail Hackett. SCCT emphasized the role of self-efficacy, outcome expectations, and personal goals in career decision-making. Incorporating cognitive and social learning principles, this theory provided a framework for understanding how individuals' beliefs and social environments influence their career management (Lent et al., 1994).

## 2 How the Research Topic contributes to understanding social constructionism in the field of career development

Four papers represent a multifaceted exploration of the dynamic interplay between personal and environmental characteristics, as well as social construction mechanisms across diverse settings. Silva et al. investigated therapeutic collaboration within career construction counseling in Portugal, which aligns with the interest in understanding how social constructionism operates within counseling contexts. By analyzing the collaborative process between counselors and clients, this study provides insights into how meanings and narratives are constructed and negotiated in career dialogues and career-support relationships. Leung explored how CCT could inform the development of a computer-assisted career guidance system that applied this theory to technology-mediated interventions. The efficacy of a digital system developed in Hong Kong, called Infinity, was examined in terms of decision-making difficulties, decision clarity, and career planning. To highlight the importance of contextual factors in shaping individuals' career behaviors, Di Maggio et al. investigated how cognitive priming related to the pandemic influenced Italian teachers' tendencies toward sustainability and a less conservative socioeconomic vision. By examining the mediating roles of state anxiety and personal need-for-structure, their study extended the current understanding of the psychological mechanisms that can drive career attitudes and decision-making. Lee and Jung emphasized Korean individuals' adaptation to today's changing environment and concentrated on how university students cognitively regulate their emotions and how these strategies affect their career decision-making self-efficacy. This study contributes to our understanding of the cognitive processes involved in shaping career development.

While these four studies explored the spectrum of social constructionism differently, central to their pursuit is the recognition of social construction mechanisms as forces influencing career trajectories in diverse societal and cultural contexts. Understanding how these mechanisms operate can provide insights into how individuals develop careers in various settings and the implications they have for career counseling.

## 3 Recommendations for ongoing endeavors in the study of social constructionism

Despite Young and Collin's (2004) argument two decades ago, significant progress has not been made. The findings have been accumulated separately rather than integrally over these years, much like the work done on our Research Topic. Continuous efforts are essential to advance social constructionism. At the core of this endeavor to advance the study of social constructionism in the field of career management lies the empowerment of individuals' career agency and motivation (Chen, 2006, 2015; Chen and Hong, 2020a,b). By examining the interplay among personal beliefs, societal expectations, and social networks, researchers can examine the mechanisms driving individuals to pursue and persist in their chosen career paths. This exploration helps to explain how career motivation operates between internal agency and external influences, shedding light on the complexities of career decision-making processes.

Another key aspect of these ongoing studies is the construction of goals and the strategic actions taken to achieve them. Investigating how individuals perform goal-directed actions during their career pursuits provides insight into the process of career construction (Young and Valach, 2004). By examining the strategies employed to cope with barriers encountered, researchers can explain the pathways through which individuals shape their career identities and aspirations within their real-life environments.

Meaning-making processes play a fundamental role in career difficulties (Chen, 2011; Kogan, 1998). Through the lens of social constructionism, future studies could explore how individuals construct narratives to understand the challenges faced in their careers (Cochran, 1990; Maree, 2019). By understanding the effective ways in which personal meanings are generated through self-reflection and dialogue regarding one's career with others, career practitioners can provide tailored support and interventions to facilitate resilience and growth.

Reflective practices, both as dialogue and through dialogue, have emerged as essential tools in the contemporary career management process (Hermans, 1987, 1999; McIlveen and Patton, 2007; Reid et al., 2016). Engaging individuals in reflective dialogue with themselves and others fosters self-awareness, insight, and clarity in career decision-making. They also contribute to the initiation, maintenance, and enhancement of career motivation. In particular, facilitating reflective practices within counseling settings allows counselees to explore their career narratives, challenge underlying assumptions, and explore alternative paths.

In addition to career dialogues, effective career feedback serves as a catalyst for career learning, growth, and development. By providing constructive feedback grounded in social constructionist principles (Van den Bergh et al., 2014), career counselors, mentors, and coaches can guide individuals toward career self-management with greater self-efficacy and confidence in their career choices. Knowing how to provide useful feedback facilitates career practitioners' understanding of their counselees' strengths, values, and aspirations, thus paving the way for informed career decisions.

Finally, innovative techniques such as career writing (Lengelle and Meijers, 2014; Lengelle et al., 2013, 2014), self-confrontation (Young et al., 1994), video playback (Young et al., 1994), and the use of images, collages, and photographs (Chan et al., 2012; Chant, 2020; Ginicola et al., 2012), as well as digital storytelling (Wu and Chen, 2020), provide alternative avenues for self-reflection and narrative construction in career exploration, decision making, and management. These creative approaches allow individuals to externalize and reframe career-related problems (Chan et al., 2012; Ricks et al., 2014) and foster new perspectives and insights into career learning and development. The establishment of these techniques will contribute to the study and practice of social constructionism in the field of career development.
